# Chronic atrial and intestinal dysrythmia syndrome: A late‐onset intestinal pseudo‐obstruction and cardiac dysfunction due to an SGO1 mutation

**DOI:** 10.1002/jpr3.70060

**Published:** 2025-07-03

**Authors:** Linda Adouane, Pierre Poinsot, Julie Castilloux, Dorothée Dal Soglio, Christophe Faure

**Affiliations:** ^1^ Department of Pediatrics, Division of Pediatric Gastroenterology, Hepatology, and Nutrition Centre Hospitalier Universitaire Sainte‐Justine, Université de Montréal Montréal Québec Canada; ^2^ Division of Pediatric Gastroenterology, Hepatology, and Nutrition CHU de Québec, Université Laval Québec Québec Canada; ^3^ Department of Pathology Centre Hospitalier Universitaire Sainte‐Justine, Université de Montréal Montréal Québec Canada

**Keywords:** CAID syndrome, dysrhythmia, gut motility, interstitial cells of Cajal, pediatric intestinal pseudo‐obstruction, sinus dysfunction

## Abstract

**Objectives:**

Pediatric intestinal pseudo‐obstruction (PIPO) is a rare, heterogeneous, and severe gut motility disorder. In 2014, Chetaille et al. described chronic atrial and intestinal dysrhythmia (CAID) syndrome associated with a recessive SGO1 mutation (p.Lys23Glu) linking it to both intestinal pseudo‐obstruction and cardiac dysrhythmia. This study aimed to describe the clinical and nutritional features and outcomes of pediatric patients with the homozygous SGO1 (p.Lys23Glu) mutation.

**Methods:**

We retrospectively enrolled children under 18 years with PIPO and homozygous (p.Lys23Glu) SGO1 mutation.

**Results:**

Eight patients were included (five girls), two were first‐degree relatives. All exhibited a typical PIPO clinical presentation, but with a later onset than usually seen in primary PIPO (median age: 6.3 years). Contrast studies revealed massively distended small bowel and colon in all patients. Antroduodenal and/or colonic manometry, performed in six patients, revealed a neuropathic pattern. A full‐thickness intestinal biopsy showed variable fibrosis of the smooth muscle internal layer associated with an abnormal presence of Cajal cells within the muscular layers. All patients required parenteral nutrition (PN) at a median age of 11.8 years. Five patients needed an ileostomy. At 18 years, one patient was off PN. Three patients developed sinus dysfunction: one at the time of PIPO diagnosis and two later. All required pacemakers. One patient died in her twenties secondary to cardiac complications.

**Conclusions:**

Recessive SGO1 mutation is a form of late onset PIPO associated with sinus dysfunction. Long‐term outcome is unclear. Cardiac, intestinal, and neurologic follow‐up is recommended as cerebral small vessel disease has been described in adults.

## Abstract (highlights)

**What is Known:**

- In 2014, the chronic atrial and intestinal dysrhythmia (CAID) syndrome associated with a recessive SGO1 mutation was decribed as a progressive failure of pacemaking tissues in both gastrointestinal and cardiac systems.

**What is New:**

- Children exhibit a typical pediatric intestinal pseudo‐obstruction (PIPO) clinical presentation but with a later onset than usually seen in primary PIPO.
- We recommend testing for SGO1 mutations in children presenting with (1) PIPO with sinus dysfunction (bradycardia), (2) isolated PIPO, or (3) sinus dysfunction associated with symptoms of intestinal dysmotility.
- Cerebral small vessel disease has been described in adults and supports the need of neurologic follow‐up in patients with SGO1 mutation.

## 1 INTRODUCTION

Pediatric intestinal pseudo‐obstruction (PIPO) is a rare, heterogeneous, and severe gut motility disorder characterized by symptoms of intestinal obstruction in the absence of a mechanical cause. It results from developmental and pathological processes, which affect the intrinsic or extrinsic intestinal neurons, intestinal smooth muscle or interstitial cells of Cajal.^1^

Advances in genetic sequencing have allowed identification of multiple mutations associated with PIPO, enabling genetic confirmation in patients with clinically suggestive symptoms.^2^ This genetic confirmation is part of the diagnostic criteria suggested by the European Society for Pediatric Gastroenterology, Hepatology, and Nutrition led expert group, which revisited PIPO diagnostic criteria in 2018.^3^

In 2014, Chetaille et al. identified a new form of chronic intestinal pseudo‐obstruction, termed chronic atrial and intestinal dysrhythmia (CAID) syndrome which is associated with a recessive SGO1 mutation (p.Lys23Glu). This mutation represents a novel cohesinopathy affecting both gut and cardiac rhythm.^4^ The present study reports a case series describing the clinical presentation, specific digestive and cardiac features, and follow‐up of pediatric patients with the SGO1 mutation. The objective is to characterize the phenotype of patients with this mutation, detailing their digestive symptoms, disease‐specific complications, treatments, and outcomes.

## 2 METHODS

This single‐center retrospective study was conducted in the Pediatric Gastroenterology‐Hepatology‐Nutrition division of CHU Sainte‐Justine in Montreal, Canada in 2023. All patients under 18 years of age diagnosed with SGO1‐mutated PIPO were included in this study. Written informed consent was obtained from all patients and their caregivers. All data were collected retrospectively from patients' files. Descriptive data are expressed as median and range. Full intestinal sections were obtained for histopathological analysis, and specific staining techniques were applied: hematoxylin eosin‐safran (HES), Masson Trichrome staining (fibrosis), immunostaining for CD117 (interstitial Cajal cells and mast cells), alpha smooth muscle actin (smooth muscle cells) and calretinin (enteric neurons and nerve fibers). Toluidine Blue was performed for the detection of mast cells. Case findings were compared with a normal full‐thickness ileal specimen from a 13‐year‐old girl who underwent surgery for ileocecal Crohn's disease.

### 2.1 Ethics statement

Our study was reviewed by CHU Sainte Justine's ethical board and didn't require approval.

## 3 RESULTS

### 3.1 Patients

We included eight patients (five female), seven of whom were French‐Canadian, with one patient of First Nations descent. Two were first‐degree relatives. Demographic, clinical, and imaging characteristics are summarized in Table 1.

**Table 1:** Patients.

Characteristics	Total (*n* = 8)
Female	5 (63%)
Family history	2 (25%)
Prenatal history	0 (0%)
Symptoms	
▪ GI	8 (100%)
▪ Cardiac (sinusal dysfunction/bradycardia)	3 (37.5%)
▪ Other (neurologic, urological)	0 (0%)
Age of symptoms onset (year) (median, range)	6.3 (2–15.6)
Age at diagnosis (year) (median, range)	7.8 (4–15.7)
Time from onset symptoms to diagnosis (months) (median, range)	10.25 (0.5–53.5)
Diagnosis based on GI symptoms only	7 (87.5%)
Diagnosis based on cardiac symptoms only	0 (0%)
Diagnosis based on both GI and cardiac symptoms	1 (12.5%)

Abbreviation: GI, gastrointestinal.

There was no family history of gut dysmotility or cardiac dysrhythmia except in the girl whose brother was affected.

All patients had a typical clinical presentation of PIPO with a variable association of abdominal distension, episodes of subocclusion, and constipation at a median age of 6.3 years (2–15.6 years). The median age at diagnosis was 7.8 years (4–15.7 years). At presentation, none of them had any neurological or urological symptoms. Seven patients were diagnosed with gastrointestinal (GI) presentation; one was referred by a cardiologist as he had both GI and cardiac symptoms.

Three patients developed cardiac manifestations (dysrhythmia from sinus or atrial dysfunction) such as severe bradycardia (clinical and on electrocardiogram): one at the time of PIPO diagnosis and two during follow‐up. All required a pacemaker at a median time of 4 years (0.5–5.2) after diagnosis.

**Figure 1:**
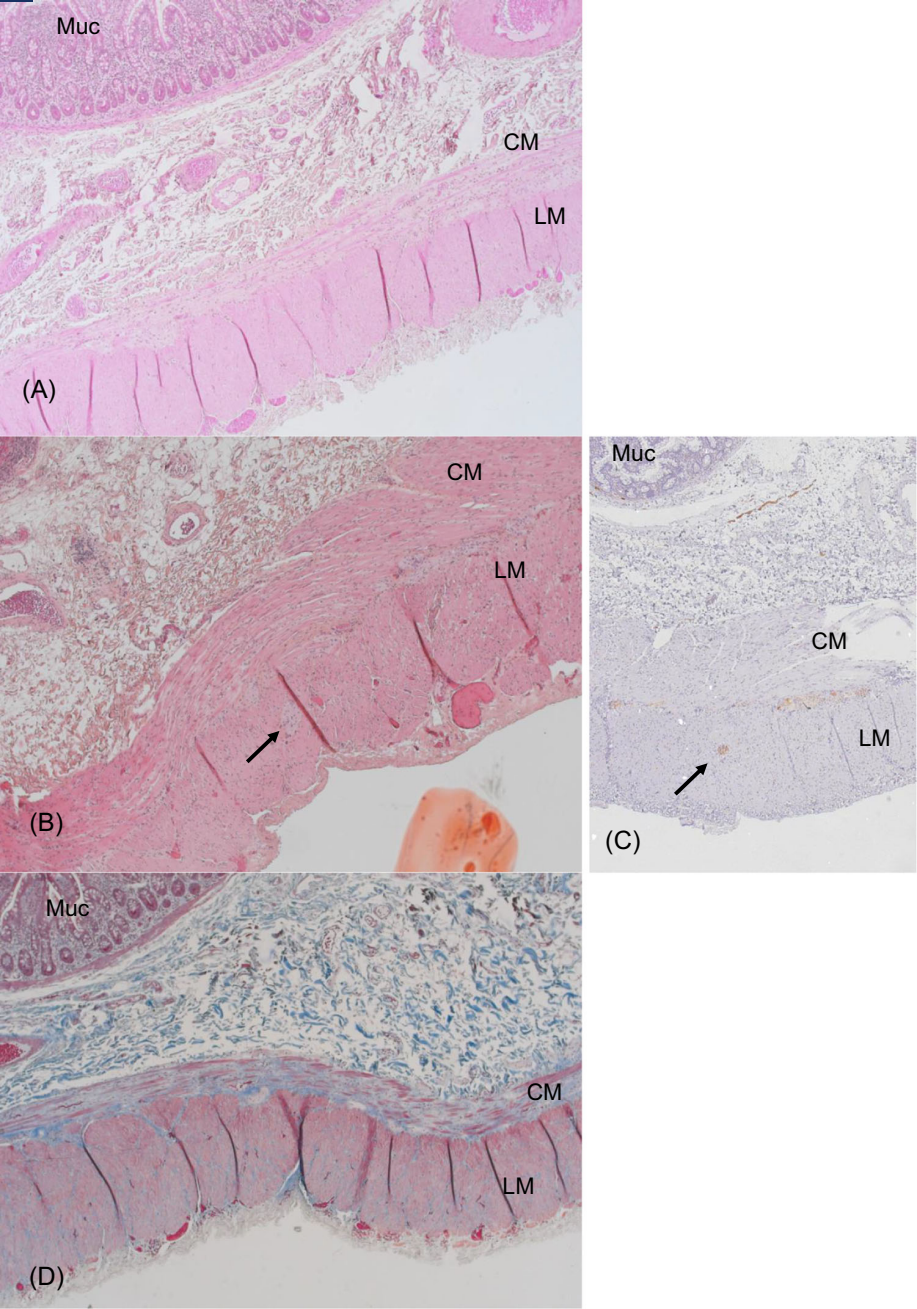
(A, B) Full thickness ileal biopsy (H&ES staining) in two CAID patients. (C) Calretinin staining in patient B. (D) Masson trichrome staining in patient A. Extensive fibrosis of the internal muscle layer (A, D). Note that the fibrosis is minimal in patient B. Mislocalization of nerve fibers in the external muscle layer (arrow) in patient B confirmed by the calretinin staining (arrow in C). (magnification ×50). CAID, chronic atrial and intestinal dysrhythmia; CM, circular muscle layer; H&ES, hematoxylin eosin‐safran; LM, longitudinal muscle layer; Muc, mucosa.

**Figure 2:**
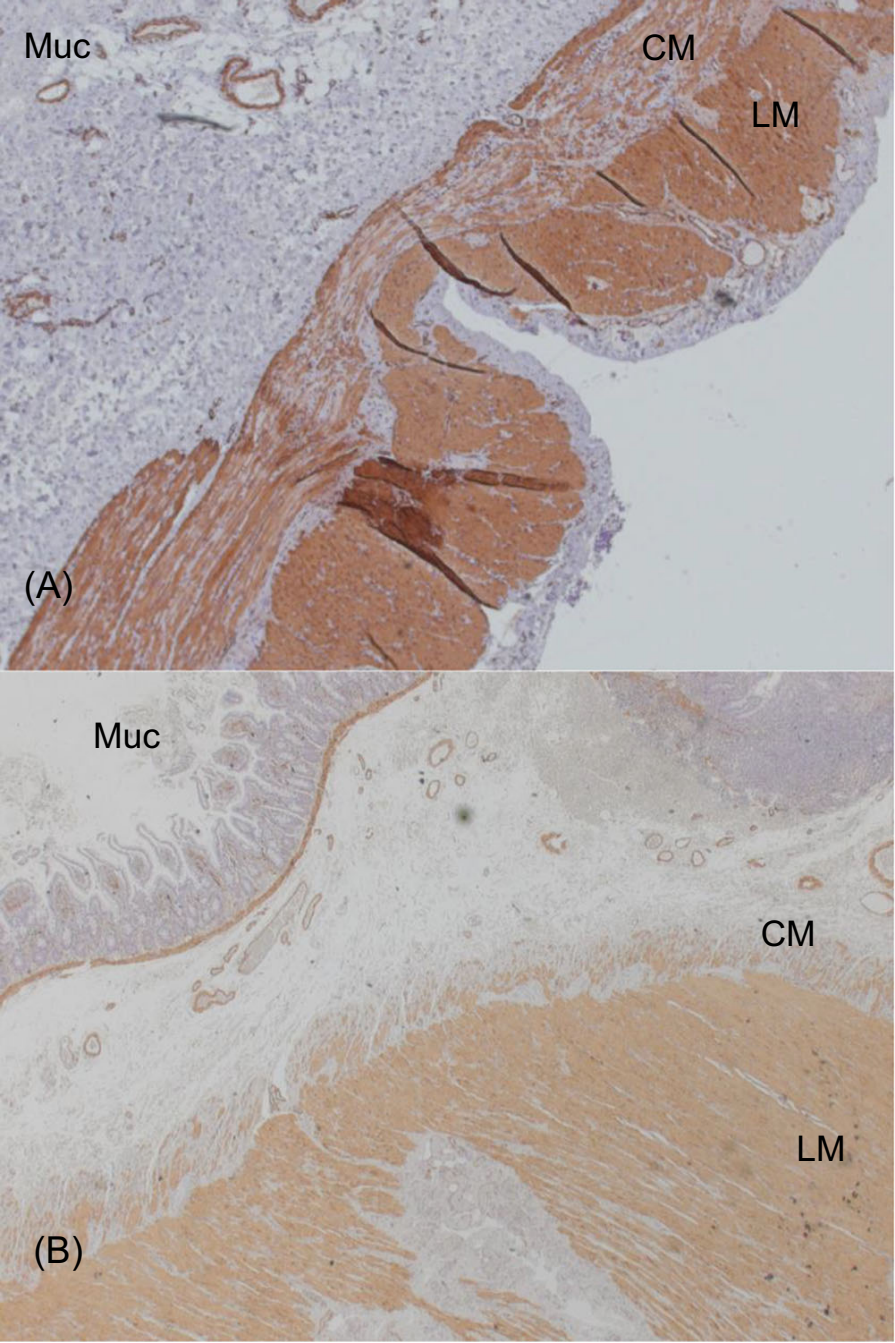
Full thickness ileal biopsy in two CAID patients; immunostaining for alpha smooth muscle actin (smooth muscle cells) showing vanishing of internal layer smooth muscle cells (A: magnification ×50; B: magnification ×25). CAID, chronic atrial and intestinal dysrhythmia; CM, circular muscle layer; LM, longitudinal muscle layer; Muc, mucosa.

**Figure 3:**
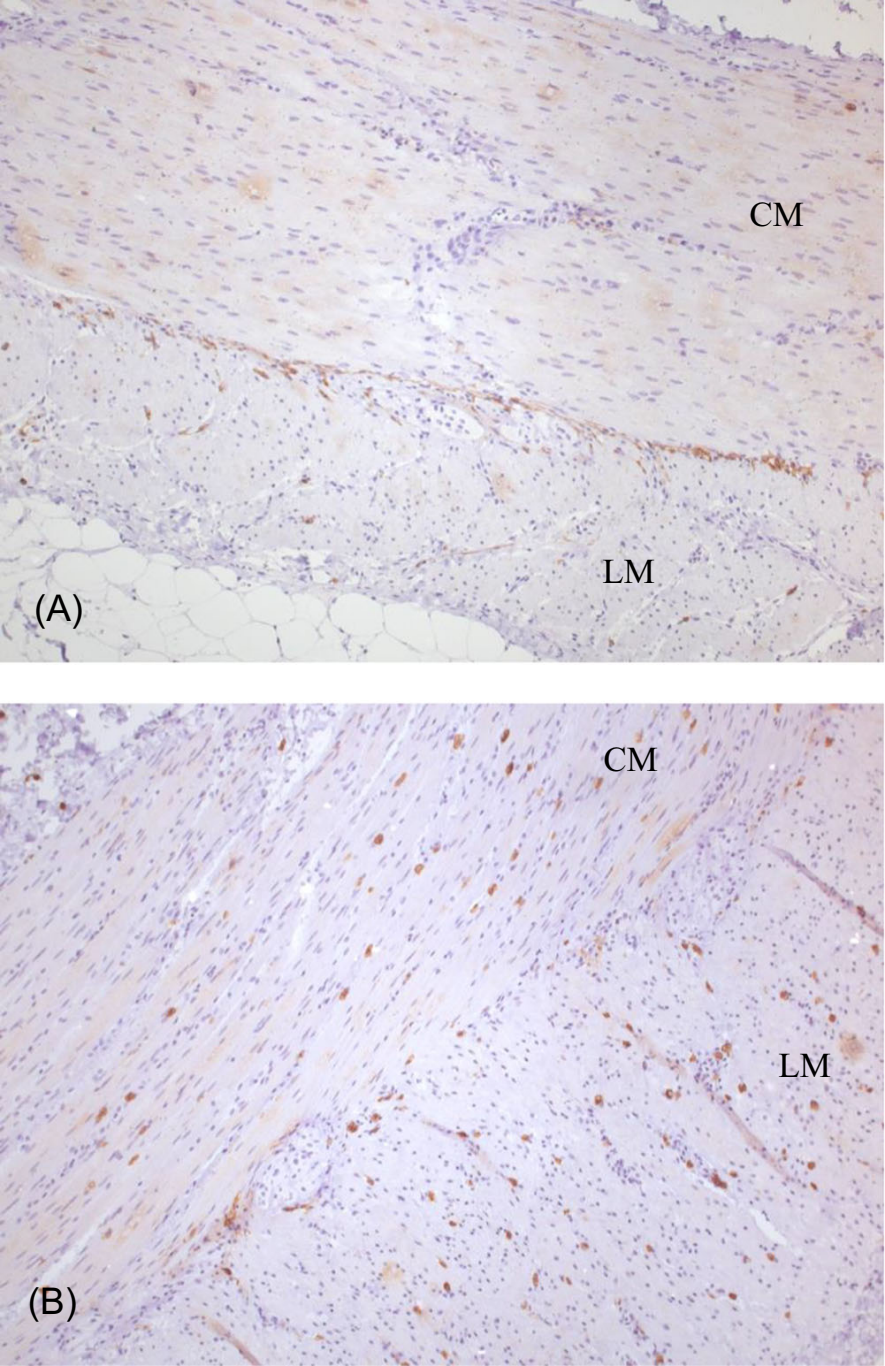
Full thickness ileal biopsy; immunostaining for CD117 (interstitial cells of Cajal) in a control child (A) operated for ileocecal Crohn's disease and (B) a CAID patient. Mislocalization of interstitial cells of Cajal within the muscular layers in CAID syndrome (B) whereas ICCs are located around myenteric plexi in control specimen (A) (magnification ×100). CAID, chronic atrial and intestinal dysrhythmia; CM, circular muscle layer; LM, longitudinal muscle layer.

Contrast studies in all patients revealed massively distended small bowel and colon without any signs of mechanical obstruction (Figure S1). Antroduodenal and/or colonic manometry, performed in six patients, revealed an abnormal neuropathic pattern (Figure S2). Full‐thickness intestinal biopsy was obtained in three patients and showed variable but sometimes extensive fibrosis of the smooth muscle internal layer which did not correlate with the intensity of symptoms (Figure 1). Immunostaining for smooth muscle actin confirmed the destruction of the internal muscle layer (Figure 2). This was associated with an abnormal presence of Cajal cells within the muscular layers (Figure 3). Toluidine blue staining confirmed that mast cells were confined to the mucosa, with none found within the muscle layers (not shown). Calretinin staining was normal and showed normal ganglia and neurites projections within the intestinal mucosa with mislocalization of nerve fibers within external muscular layer (arrows in Figure 1B,C).

SGO1 mutation (p.Lys23Glu) was identified retrospectively in four patients as the CAID syndrome had not yet been discovered when these patients were initially diagnosed with PIPO.

Four additional patients were diagnosed based on the combination of digestive symptoms of intestinal pseudo‐obstruction and the presence of the SGO1 (p.Lys23Glu) mutation. Among these, one patient also exhibited sinus dysfunction at the time of diagnosis.

### 3.2 Treatment

All patients required parenteral nutrition (PN) at a median age of 11.8 years (range: 6.5–15.9 years), approximately 2.8 years (range: 0.02–7.1 years) after diagnosis, due to failure to thrive, intestinal failure, and electrolyte disorders. All patients were prescribed prokinetics (domperidone, cisapride, prucalopride, and metoclopramide were mostly prescribed) while antibiotics (cycled amoxicillin‐clavulanic acid, metronidazole, ciprofloxacin and rifaximin, alone, or combined) for small intestinal bacterial overgrowth (SIBO) were administered to six patients. Five patients underwent ileostomy at a median of 2.6 years (range: 1.2–11.2 years) post‐diagnosis, and three patients also had a gastrostomy (Table S1).

### 3.3 Evolution

All but one patient remained PN‐dependent, with one patient successfully weaned off PN at 18 years of age. The median age of patients in this cohort was 22 years (range: 9.3–27 years. Prokinetics were not effective, regardless of the agent used, and most patients ceased them after a few months. Likewise, most of the antibiotics used to treat SIBO have shown lack of long‐term effectiveness (one patient still uses some on a cyclic basis though, metronidazole 10 days/month). They were prescribed mainly during the exacerbations of distension. One patient, who had not undergone ileostomy, developed a small bowel volvulus necessitating a small bowel resection and terminal ileostomy. One patient developed a gastric volvulus. Three patients presented a stoma prolapse. One patient died in her twenties, not directly related to PIPO but secondary to cardiac complications at 24 years. She presented with recurrent pericarditis, flutter, atrial fibrillation, pacemaker surgeries, and a cardiac tamponade. She died of a nosocomial pneumonia 2 weeks later.

None of the patients had any neurologic or urologic complications. Consequences and complications of sinus dysfunction (essentially bradycardia) in three of our patients were prevented with the implementation of a pacemaker. Annual EKG and cardiac follow‐up are carried out.

## 4 DISCUSSION

CAID syndrome is a progressive disorder characterized by the failure of pacemaking tissues in both GI (interstitial cells of Cajal) and cardiac (sinoatrial node) systems.^4^ Patients share a combination of two disorders: the sick sinus syndrome and intestinal pseudo‐obstruction. It is due to an autosomal recessive mutation of SGO1 (p.Lys23Glu) described by Chetaille et al. in 2014 in 17 patients (16 French‐Canadians and 1 Swedish).^4^ Due to the founder effect observed in the province of Quebec, Canada, we had the opportunity to follow a large cohort of children with CAID syndrome. We, therefore, describe here the first pediatric series of CAID syndrome with their clinical phenotype and follow‐up. All patients had a typical clinical presentation of PIPO but with a later onset than usually seen in primary PIPO.^5^

In their seminal paper, Chetaille et al stated that the disease‐causing haplotype was of northern European origin, shared among French–Canadian CAID cases. They identified a founder couple, married in France in 1620, whose likelihood of being the mutation carriers.^4^ More recently, Venkatesh et al. published a case‐series of two brothers aged 6 and 12 years, from the North indian Baniya community who presented with the same mutation as described by Chetaille et al. (p.Lys23Glu).^6^ A recent preprint reports on a 10‐year‐old boy from Iran and with SGO1 (p.Lys23Glu) homozygous mutation.^7^ This suggests that the mutation is present not only in Quebec, Canada but is also present globally.

The SGO1 gene encodes a component of the cohesin complex, which bundles DNA together during all phases of the cell cycle and cell division. Mutations in SGO1 disrupt cohesin protection, potentially promoting cell senescence.^4^, ^8^ In normal tissues, SGO1 is widely expressed in the intestinal wall, in smooth muscle, interstitial cells of Cajal (ICC) and the enteric neurons.^4^ In intestinal biopsies from CAID patients, Chetaille et al. reported hypoplastic ganglia and mislocalization of enteric neurons and ICCs within the circular and longitudinal smooth muscle layers. They also described vacuolization and disruption of muscle layers.^4^ In the present study, we observed variable but sometimes extensive destruction of the internal muscle layer with accompanying fibrosis. We confirmed the mislocalization of ICCs in the muscular layers and, using Toluidine blue staining, demonstrated that ICCs were absent in the mucosa. Although we did not find ectopic localization of enteric neurons within the mucosa, we did observe abnormal presence of nerve fibers in the external muscle layer. Furthermore, we found that the degree of fibrosis in the muscular layer did not correlate with symptom severity.

Cohesinopathies, which include Cornelia de Lange syndrome, are typically associated with major neurodevelopmental deficits and short stature from birth. In contrast, CAID patients exhibit no developmental delay or short stature. Patients are healthy at birth, and the earliest symptoms usually appear around the age of 6, sometimes earlier. In this study, the median age of symptom onset was 6.3 years (range: 2–15.6 years), and the median age at diagnosis was 7.8 years (range: 4–15.7 years). Piché et al. suggest that the lack of significant mitotic consequences in CAID syndrome, unlike in other cohesinopathies, likely indicates preserved cohesin function, potentially involving other, as yet unknown, molecular mechanisms.^8^ Interestingly, a recently described syndrome of intestinal pseudo‐obstruction associated with a dominant mutation in RAD21—a cohesin complex member—also hints at a noncanonical role of cohesins.^9^

In our study, all patients exhibited a late pediatric onset of a severe form of PIPO, initially presenting with GI symptoms. Only one patient experienced both GI and cardiac symptoms at diagnosis. Most patients had no family history of gut dysmotility or cardiac dysrhythmia, except for the two siblings. Before 2014 and the identification of the SGO1 mutation, the diagnosis was considered idiopathic PIPO, with or without atrial involvement. In these cases, CAID syndrome was confirmed retrospectively through genetic testing. Following the syndrome's identification, diagnoses were made based on clinical presentation and subsequently confirmed by genetic testing. None of these patients required major invasive procedures, in line with ESPGHAN expert recommendations.^3^

All patients, except one, remain dependent on PN and underwent ileostomy at around 18 years of age. This outcome is consistent with other PIPO syndromes.^1^, ^5^ None experienced cardiac complications during pediatric follow‐up. However, one patient, aged 24, developed severe cardiac complications, including recurrent pericarditis, atrial flutter, atrial fibrillation, pacemaker surgeries, and cardiac tamponade, and ultimately died from nosocomial pneumonia.

It is noteworthy to underline that Nheme et al. reported two patients with CAID syndrome who, in their 40s, presented with cerebral small vessel disease (CSVD) characterized by an unusual pattern of predominantly cerebellar microbleed development.^10^ Neurological manifestations of CAID syndrome had not previously been documented. None of our patients exhibited any neurological symptoms. Two patients had a cerebral MRI both of which was normal. One patient, however, had an abnormal MRI at the age of 20, showing cerebral microangiopathy with preferential localization in the cerebellar hemispheres and medulla. Given these findings, cerebral MRI should be part of the follow‐up and management of patients with CAID syndrome, especially if anticoagulant therapy is considered.

Although CAID syndrome is a rare condition seemingly concentrated in founder clusters, we recommend testing for SGO1 mutations in children presenting with (1) PIPO with sinus dysfunction (bradycardia), (2) isolated PIPO, or (3) sinus dysfunction associated with symptoms of intestinal dysmotility.^11^ Whether the phenotype could extend to include less severe intestinal dysmotility or severe constipation is currently unknown and warrants further investigation.

Therapy of CAID syndrome is identical to any PIPO and based on nutrition, pharmacologic, and surgical intervention. It requires a specialized multidisciplinary approach and management ^12^

Long‐term follow‐up is mandatory as the long‐term outcome is not yet well known.

## 5 CONCLUSION

The recessive SGO1 mutation represents a newly identified cohesinopathy impacting gut and cardiac rhythms, manifesting as a late‐onset yet severe form of PIPO in pediatric patients. GI symptoms (vomiting, abdominal pain, and distension) appear first and can be associated with severe bradycardia. While CAID syndrome has been described in adults, its presentation and progression in pediatrics remain unclear. The long‐term phenotype of SGO1 mutation patients is still unknown. Neurological symptoms such as CSVD have been described in adults supporting the need of a multidisciplinary follow‐up of patients with this mutation.

## AUTHOR CONTRIBUTIONS

Linda Adouane participated in data collection and analysis, drafted the initial manuscript, and approved the final manuscript. Pierre Poinsot participated in the data collection. Julie Castilloux participated in the recruitment of patients and critically revised the manuscript. Dorothée Dal Soglio revised the pathology data. Christophe Faure led the study, participated in the analysis and critically revised the manuscript. All authors approved the final manuscript.

## CONFLICT OF INTEREST STATEMENT

The authors declare no conflicts of interest.

## Supporting information

Supplemental Figure S1: Abdominal X‐ray showing massively distended intestinal loops in a 9‐year‐old girl diagnosed with CAID syndrome.

Supplemental Figure S2: Antroduodenal tracing in a 7‐year‐old girl showing a neuropathic pattern with an abnormal response to intraveinous erythromycin.

Supplemental Table S1: Treatment.
