# Supplementary figures and images for: Chronic atrial and intestinal dysrythmia syndrome: A late‐onset intestinal pseudo‐obstruction and cardiac dysfunction due to an SGO1 mutation
Source: JPGN Rep. 2025 Jul 3;6(4):327–33. doi: 10.1002/jpr3.70060 (PMC12611616; doi:10.1002/jpr3.70060)

Supplemental Figure S1

*
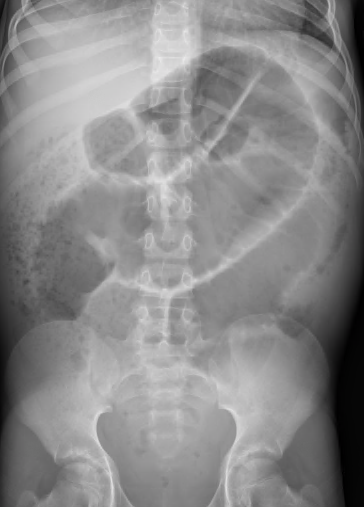
* *
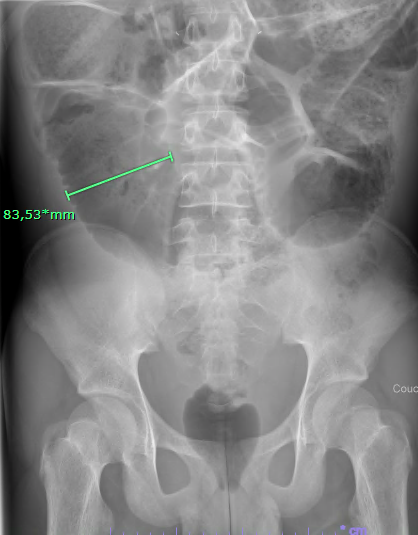
*

Supplement: Supplementary file 1 — Supplemental Figure S1: Abdominal X‐ray showing massively distended intestinal loops in a 9‐year‐old girl diagnosed with CAID syndrome. [file JPR3-6-327-s002.docx]
