# Supplementary material for: Chronic atrial and intestinal dysrythmia syndrome: A late‐onset intestinal pseudo‐obstruction and cardiac dysfunction due to an SGO1 mutation
Source: JPGN Rep. 2025 Jul 3;6(4):327–33. doi: 10.1002/jpr3.70060 (PMC12611616; doi:10.1002/jpr3.70060)
Supplement: Supplementary file 2 — Supplemental Figure S2: Antroduodenal tracing in a 7‐year‐old girl showing a neuropathic pattern with an abnormal response to intraveinous erythromycin. [file JPR3-6-327-s003.docx]

Supplemental Figure S2.

Duo

Duo

Duo

Duo

Jej

Jej

Jej

Jej
