# Supplementary material for: Chronic atrial and intestinal dysrythmia syndrome: A late‐onset intestinal pseudo‐obstruction and cardiac dysfunction due to an SGO1 mutation
Source: JPGN Rep. 2025 Jul 3;6(4):327–33. doi: 10.1002/jpr3.70060 (PMC12611616; doi:10.1002/jpr3.70060)
Supplement: Supplementary file 3 — Supplemental Table S1: Treatment. [file JPR3-6-327-s001.docx]

Supplemental Table S1

| **Treatment** | **Total (n = 8)** |
| --- | --- |
| **Nutritional**   - Parenteral Nutrition (PN) - Time from onset symptoms and start of PN (year) (median, range) - Time from diagnosis and start of PN (year) (median, range) | 8 (100%)  4.5 [0.3-7.5]  1.75 [0.2-7.1] |
| **Medical**   - Prokinetics - Antibiotics (on/off for bacterial overgrowth) | 8 (100%)  6 (75%) |
| **Surgical**   - Gastrostomy - Ileostomy - Time from onset symptoms and ileostomy (year) (median, range) | 3 (37.5%)  5 (62.5%)  2.6 [1.2-11.2] |
| **Cardiac**   - Pacemaker | 3 (37.5%) |
